# Autonomous motivation: school leaders as key drivers of physical activity in the Global Health Program

**DOI:** 10.3389/fpsyg.2025.1646775

**Published:** 2025-08-06

**Authors:** R. F. Guimaraes, J. Bélisle, B. Lamy, R. Gaudet, I. Doré, P. L. Yao, T. A. Barnett, N. Carbonneau, S. Girard

**Affiliations:** ^1^Department of Physical Activity Sciences, Université du Québec à Trois-Rivières, Trois-Rivières, QC, Canada; ^2^School of Kinesiology and Physical Activity Sciences, Faculty of Medicine, Université de Montréal, Montreal, QC, Canada; ^3^School of Public Health, Université de Montréal, Montreal, QC, Canada; ^4^University of Montreal Hospital Research Centre, Montreal, QC, Canada; ^5^Department of Family Medicine, McGill University, Montreal, QC, Canada; ^6^CHU Sainte-Justine Azrieli Research Centre, Montreal, QC, Canada; ^7^Department of Psychology, Université du Québec à Trois-Rivières, Trois-Rivières, QC, Canada

**Keywords:** motivation, physical activity, school leaders, intervention, children, adolescents

## Abstract

**Introduction:**

Motivation is one of the main factors that can influence physical activity practice in youth. Schools are ideal settings to provide opportunities to be active. However, few school-based behavioral change interventions have been designed with curriculum-based contents and evaluated in a real-life setting. The Global Health Program (GHP), implemented among 10,000 students in Québec (Canada), aims to promote long-term behavior change through educational strategies guided by school leaders. Therefore, the aim of the present study was to (i) investigate the association between school leaders’ implication and motivation for physical activity in GHP participants, and (ii) to test whether this association is moderated by physical activity level (active vs. inactive) or number of years of participation in the program.

**Methods:**

A cross-sectional study among children and adolescents participating in the GHP was conducted. The data collection was carried out in the Fall 2024 using self-report online questionnaires. Demographic data, motivation for physical activity, perception of school leaders’ implication and physical activity level were collected. Linear regressions analysis with interaction terms to examine potential moderating effect were used.

**Results:**

Results showed that among the 658 participants (42% girls, mean ± SD age = 14.5 ± 1.8 years) 29.4% reported being active participants have been involved in GHP for an average of 3.1 ± 2.1 years. There was a positive association between school leaders’ implication and autonomous motivation [ $\hat{\beta }$ = 0.26; (95%CI 0.138; 0.256)]. However, neither PA level [ $\hat{\beta }$ = − 0.02, (95%CI −0.142; 0.104)] nor years of participation in GHP [ $\hat{\beta }$ = − 0.02, (95%CI −0.144; 0.102)] moderated this relationship.

**Conclusion:**

Results support the importance of the role of school leaders on students’ motivational quality, regardless of their PA status or exposure length to intervention programs. This insight emphasizes the value of cultivating supportive school environments and leadership practices that consistently promote autonomous motivation, thereby encouraging long-term engagement in physical activity among youth, as fostered by the GHP in Québec (Canada).

## Introduction

Physical activity (PA) plays a key role in the development of children and adolescents. The Canadian 24-Hour Movement Guidelines for Children and Youth recommends that children and adolescents aged 5 to 17 years old engage in an average of 60 min of moderate to vigorous PA (MVPA) per day (Tremblay et al., 2016). Regular practice of PA is associated with multiple physical, mental and social health benefits (Oberle et al., 2025; Kuzik et al., 2025). Despite the recommendations and the many benefits associated with regular PA practice, 80% of adolescents aged 11 to 17 are not enough physically active (Guthold et al., 2020). Similarly, in Quebec (Canada), only one in five children and adolescents are sufficiently active during their leisure time (Contreras and Joubert, 2022). These results underscore the need for action to motivate youth to engage in PA.

Several external factors may explain why young people do not meet the PA recommendations. These include increase in screen time, reduced opportunities for active transportation, limited access to green spaces conducive to play, financial constraints and lack of time due to overscheduled routines (ParticipACTION, 2024). Social influences also need to be considered, such as peers through encouragement and appreciation (Manyanga et al., 2024). In addition, caregivers, such as teachers or coaches, can play a decisive role in creating an inclusive, stimulating and caring motivational climate (Girard et al., 2023). However, internal factors can also influence PA. For instance, the pleasure, sense of efficacy or interest associated with an activity can directly influence the desire to be physically active (Cachón-Zagalaz et al., 2023; Qi et al., 2024). Indeed, motivation represents an important lever for understanding why some young people are physically active or not.

**Figure 1:**
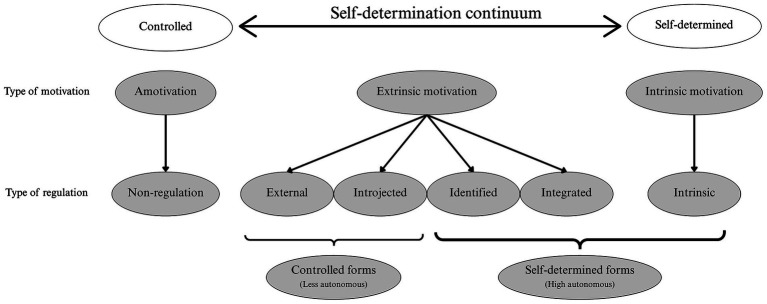
Self-determination continuum by Ryan and Deci (2000). Reproduced with permission from the American Psychological Association. No further reproduction or distribution is permitted.

Self-determination theory (Ryan and Deci, 2000) explains that satisfaction of the basic psychological needs (autonomy, competence and relatedness) is a necessary condition for psychological development, integrity and well-being. Ryan and Deci (2000) conceptualize self-determination as a continuum of motivation, which includes amotivation, extrinsic and intrinsic motivation. These three overarching categories can be further differentiated into six types of regulation, as illustrated in Figure 1.

On the left, amotivation refers to the absence of intention to act (i.e., no perceived link between action and results). In the center, extrinsic motivation (when the person performs an action to attain a separable outcome, such as a reward, recognition, or to avoid punishment) is separated in two: controlled (comprising external and introjected regulation), and self-determined forms (comprising identified, and integrated regulation). On the right, intrinsic motivation refers to the pleasure, curiosity or personal interest towards the activity. These three types of motivation are distinguished by different types of regulation, which reflect the degree of autonomy perceived by the individual in behavior, influencing the quality of engagement in the activity (Ryan and Deci, 2000). Specifically, external regulation occurs when the individual acts to avoid a consequence or obtain a reward, under external pressure. Introjected regulation, on the other hand, comes from internal pressure: the individual acts to avoid feeling guilty or anxious, or to avoid damaging his/her self-esteem. As for self-determined forms, identified regulation occurs when the individual recognizes that the activity is important to him to achieve personal goals, so he/she participates voluntarily, even if it is not always pleasant. Integrated regulation occurs when the activity makes sense to the individual and corresponds to his or her personal values.

In the school context, teachers (or school leaders) play a fundamental role in meeting students’ basic psychological needs by the motivational climate they create. In this line, the motivational climate can be need-supportive or need-thwarting (da Silva et al., 2025). When the climate supports students’ needs for autonomy, competence, and relatedness, it fosters more self-determined forms of motivation. Conversely, when the climate thwarts these needs, it tends to promote more controlled forms of motivation. In short, the motivational climate created by school leaders can be a key factor that, when need-supportive, can encourage a shift in student motivation towards the more self-determined forms of the continuum.

Schools are ideal settings to implement healthy lifestyle interventions as they provide a social environment, by enrolling the whole school community (families, school leaders and students). Also, it provides opportunities to be active (recess, physical education, physically active lessons and outdoor classes) and can emphasize the PA promotion in educational curricula. However, few school-based behavioral change interventions have been designed with curriculum-based contents and evaluated in a real-life setting (Ourda et al., 2025).

Several school-based intervention programs have been implemented across different countries to promote healthy lifestyles among children and adolescents, with varying degrees of success. However, most school-based interventions focused on single-movement behaviors (such as PA, sedentary behavior, or sleep) have yielded non-significant or limited effects (Rodrigo-Sanjoaquín et al., 2025). An overflow but small effect on non-targeted behaviors was found in interventions targeting only PA and sedentary behavior (Rodrigo-Sanjoaquín et al., 2025). Most of the programs offers extracurricular activities and they do not address the need to optimize time. A recent review (da Silva et al., 2025) recommended that interventions should prioritize long-term set, enhance student-teacher relationship and collaboration, increase the level of interest in physical education and the participation in enjoyable activities (as outdoors), develop young people’s knowledge, and address contextual factors affecting PA. Also, a whole-school approach is needed to increase student PA (Ourda et al., 2025).

The Global Health Program (GHP), intervenes among 10,000 students in Québec (Canada), guiding them toward long-term behavior change through two educational content strategies that will be detailed further.

Interventions are made by people already working in schools, primarily teachers, who receive training and tools from the program (the school leaders). It is one of the first initiatives in Quebec that can impact motivation and is integrated into classroom hours. This is why GHP initiative stands out from the programs mentioned. In addition, while programs elsewhere in the world have been evaluated, most programs implemented in Quebec schools have not, except for FitSpirit (Leduc et al., 2021; Ahmadi et al., 2025; Guimarães et al., 2021; Guimarães et al., 2022; Paiement et al., 2020) (an extracurricular girls-only intervention). Conducting a rigorous and detailed evaluation of the GHP, an initiative intended for all students in participating schools, rather than a specific subgroup or a limited number of participants, appears essential to determine the levels at which it is effective and whether it meets the intended objectives.

Given all aforementioned gaps, the inconsistent findings and the importance of evaluating the extent to which the GHP can benefits these students’ lifestyle behaviors, the aim of the present study is to (i) investigate the association between school leaders’ implication and motivation for PA in GHP participants, (ii) to test whether this association is moderated by PA level (active *vs* inactive) or number of years of participation in the program; and (iii) to explore how different types of motivation for PA are associated with PA level. We hypothesized that (i) autonomous motivation for PA will be associated with positive school leader skills; (ii) the association will be stronger among those who are physically active compared to those who are inactive, and those with greater number of years of participation in the program; and (iii) identified and intrinsic motivation will be associated with higher PA level.

## Materials and methods

### Study design

The cross-sectional data were drawn from the Autumn 2024 baseline evaluation of the GHP quasi-experimental study. The GHP^1^ is delivered among approximately 10,000 students from 48 public schools distributed in 9 administrative regions of Quebec (Canada). GHP aims to guide students toward long-term behavior change through two educational content strategies: (i) offering outdoor-oriented activities and (ii) developing components integrated into school subjects (nutrition, life balance and stress management, human body, and first aid). These contents are integrated into the school curriculum aiming to contribute to the students’ overall well-being.

### Participants

Baseline sample includes 658 students aged 6 to 17 years. All forty-eight schools enrolled in the GHP in Québec (Canada) were recruited and all GHP participants were invited to take part to the study. The invitation was sent by the Director of the GHP via email. Data were collected using an online questionnaire (Qualtrics) available in both French and English between September and November 2024. Participants provide electronic consent.

### Instruments

#### Demographics

Self-reported age, sex, ethnicity, parental education, participation in GHP activities (number of years, activities performed) and peers’ presence in the GHP were identified as confounders by a literature review to be considered in the regression models.

#### Motivation

The Behavioral Regulation in Exercise Questionnaire-2 (BREQ-2)^2^ ^3^ was used to document motivation for PA (Markland and Tobin, 2004). The BREQ-2 is a 19-item questionnaire assess five dimensions of the self-determination continuum using a 5-point Likert scale (0 = *not true for me*, 5 = *very true for me*): amotivation (Cronbach *α* = 0.83), external (Cronbach α = 0.79), intrinsic (Cronbach α = 0.86), identified (Cronbach α = 0.73), and introjected (Cronbach α = 0.80) regulation. Average scores were computed for each dimension and summed to generate composite scores for autonomous motivation (intrinsic regulation + identified regulation), controlled motivation (introjected regulation + external regulation) and amotivation. Relative Autonomy Index (RAI) (Chemolli and Gagné, 2014), which is the individual’s degree of autonomous motivation towards PA, was calculated using the following formula: [amotivation × (−3)] + [external regulation × (−2)] + [introjected regulation × (−1)] + (identified regulation × 2) + (intrinsic regulation × 3). The maximum score for the RAI is +20 and the minimum score is −24. Low autonomous motivation is indicated by lower negative scores on the RAI (< 0). Higher autonomous motivation is indicated by positive scores for the RAI (> 0). In this study, the index ranged from −11 to 19. The RAI is justified based upon the continuum logic in which each type of motivation is predictably ordered and can be classified as either positive or negative, as denoted by the weightings, with external and introjected regulations reducing the degree of autonomy and identified and intrinsic motives increasing the level of relative autonomy. By adding these positively and negatively weighted scores together, this index estimates the overall degree of relative autonomy and as such approximates an individuals’ position along the underlying continuum of self-determination.

### Physical activity

Participants were asked to report the amount of time spent doing each of the activities listed in the Physical Activity Questionnaire for Children or Adolescents (PAQ-C; PAQ-A) (Mestre and Reychler, 2021) for the past week. The PAQ-C is appropriate for elementary school-aged children (ages 6–14) who are currently in the school system and have recess as a regular part of their school week. The nine PAQ-C questions are organized using a segmented time-of-day or day-of-the-week strategy. It provides a summary PA score derived from nine items, each scored on a 5-point scale (Cronbach *α* = 0.79), and includes the following: In the last 7 days, what did you do most of the time at recess? (Tremblay et al., 2016) Sat down (talking, reading, doing schoolwork); (Oberle et al., 2025) Stood around or walked around; (Kuzik et al., 2025) Ran or played a little bit; (Guthold et al., 2020) Ran around and played quite a bit; (Contreras and Joubert, 2022) Ran and played hard most of the time. The PAQ-A is appropriate for high school students (approximately ages 14–20) who are currently in the school system, and it provides a summary PA score derived from eight items (no recess item), each scored on a 5-point scale, as follows: “In the last 7 d, during your physical education (PE) classes, how often were you very active (playing hard, running, jumping, throwing)?” (Tremblay et al., 2016) I do not do PE; (Oberle et al., 2025) Hardly ever; (Kuzik et al., 2025) Sometimes; (Guthold et al., 2020) Quite often; (Contreras and Joubert, 2022) Always. Responses were averaged to derive a mean score for each activity and combined to form a total PA score (between 1 and 5) (Cronbach *α* = 0.85).

### Students’ perception of school leaders’ implication

Students’ perception of school leaders’ implication on GHP was assessed using a scale including seven items developed by the researchers and the GHP director. Items were measured on a 5-point Likert scale from *strongly disagree* to *strongly agree* and includes the following: “The school leader was able to create a pleasant and enjoyable atmosphere; was dynamic and encouraged everyone’s participation during the sessions; inspires me to be physically active and have good lifestyle habits regularly.” For the descriptive analysis only, we dichotomized the final score as “Positive skills” (agree and strongly agree) and “Less positive skills (neutral, disagree and strongly disagree).

### Data analysis

Continuous and categorical variables were descriptively summarized using means and standard deviations (*SD*) and percentages. Normality of continuous variables’ distributions was checked by computing their level of skewness and inspecting their QQ-plots.

Independent *t*-test, ANOVA and Chi-square analysis were performed to examine differences in the characteristics of participants between motivation profiles. (1) Linear regression analyses crude and adjusted for potential confounders (age, sex and mother’s education) were performed to estimate the association between students’ perception of school leaders’ implication and motivation for PA; (2) motivation for PA and PA level. To test whether this association is moderated by PA level (active *vs* inactive) or number of years of participation in the program (< 2y *vs* ≥ 3y), we then include interaction terms between school leader implication*PA level and school leader implication*years of participation in separate models.

Statistical significance was defined as *p* ≤ 0.05 and 95% CI are presented. All analyses were performed using IBM SPSS (IBM SPSS Statistics, Version 28.0. Armonk, NY, USA) and the moderation analysis was run using the version 3.5 of PROCESS command (Hayes and Rockwood, 2017).

### Ethics statement

The Université du Québec à Trois-Rivières’ Board gave ethical approval for the conduct of the study (CER-24-309-07.04). Participating schools and students gave approval for conduct of research activities.

## Results

Table 1 presents the participant characteristics. Results showed that among the 658 participants (42% girls, mean ± SD age = 14.5 ± 1.8 years) 29.4% reported being active participants have been involved in GHP for an average of 3.1 ± 2.1 years. Table 1 also shows the participant characteristics according to motivation profiles: 96% were classified as reporting high autonomous motivation and 4% report low autonomous motivation. No statistically significant differences between motivation profiles were observed in years of participation in GHP, mother’s education, PA and students’ perception of program leader’s implication. However, sex (*p* = 0.001), age (*p* = 0.05) and peers’ presence in the GHP (*p* = 0.05) were different across motivation profiles. The participants with high autonomous motivation profile were younger (14.6 years) than those with low autonomous motivation (15.2 years).

**Table 1:** Characteristics of participants by motivation profile (*n* = 658).

Participant’s characteristics	Total	High autonomous motivation *n* = 625 (95.8%)	Low autonomous motivation *n* = 33 (4.2%)	*p*
Years of participation in GHP	3.1 (2.1)	3.6 (2.1)	3.4 (2.4)	
Sex, %				
Girls	42%	**98.5%** ^**a**^	**1.5%**	**0.001**
Boys	58%	**93.8%** ^**b**^	**6.2%**	
Age, mean (SD)	14.5 (1.8)	**14.6 (1.7)**	**15.2 (1.4)**	**0.05**
Mother’s education, %				
Attended college/university	90.6%	96.2%	3.8%	
Attended high school only	9.4%	93.5%	6.5%	
Physical activity, %				
Active	29.4%	94.3%	5.7%	
Inactive	70.6%	96.5%	3.5%	
Peers support, %				
Best friends in the GHP	90.5%	**97.6%** ^**a**^	**2.4%**	**0.05**
No friends in the GHP	9.5%	**93.0%** ^**b**^	**7.0%**	
School leader implication, %				
Positive skills	40.1%	98.3%	1.7%	
Less positive skills	59.9%	96.0%	4.0%	

GHP, Global Health Program. Data are presented in means (SD) or %. *PAQ-C and PAQ-A questionnaire. Scores are interpreted as follows: 1 and 2 = Inactive; 3, 4 and 5 = Active. ×Likert scale from 1 (strongly disagree) to 5 (strongly agree). Statistically significant differences between profiles at *p* < 0.05 level are bold. ^a,b^*T*-test and Chi-square tests results.

Considering the low percentage of participants classified as low autonomous motivation, further analysis was conducted using the RAI continuous variable, as the RAI is justified based upon the continuum logic, as detailed in the methods section. The mean RAI score was 12 ± 4.9 for girls and boys together.

Table 2 presents demographic data displayed by types of motivation. Older girls presented higher means of amotivation, whereas older boys showed higher means of amotivation, and external regulation compared to younger ones. Higher percentages of high autonomous motivation were found both in girls and boys. Additionally, no significant differences were found between age groups within sexes.

**Table 2:** Demographic data by types of motivation (*n* = 658).

Groups	Total (*n*)	Amotivation Mean (SD)	External regulation Mean (SD)	Introjected regulation Mean (SD)	Identified regulation Mean (SD)	Intrinsic regulation Mean (SD)	High autonomous motivation (%)	Low autonomous motivation (%)
Girls <14 y	67	**−0.4 ± 1.1** ^**a**^	−0.9 ± 1.1	−1.2 ± 1.1	5.1 ± 1.5	10.4 ± 1.8	100%	0%
Girls ≥14 y	204	**−0.5 ± 1.0** ^**a**^	−0.9 ± 1.4	−1.5 ± 1.0	5.5 ± 1.3	9.9 ± 2.0	98.0%	2.0%
Boys <14 y	86	**−0.9 ± 1.5** ^**b**^	**−0.9 ± 1.3** ^**b**^	−1.2 ± 1.0	5.3 ± 1.5	10 ± 1.9	97.7%	2.3%
Boys ≥14 y	282	**−1.3 ± 2.4** ^**b**^	**−1.4 ± 1.8** ^**b**^	−1.6 ± 1.1	5.4 ± 1.5	10 ± 2.2	92.9%	7.1%
Total		−0.9 ± 1.9	−1.1 ± 1.6	−1.5 ± 1.1	5.4 ± 1.4	10 ± 2.1	100%	0%

Types of motivation scores are presented as means and standard deviation. Average scores were computed for each dimension of the questionnaire. Statistically significant differences between types of motivation at *p* < 0.05 level are bold. ^a,b^*T*-test results between sexes by types of motivation. No significance between age groups within sexes were found. Chi-square tests results.

Table 3 shows the association of each type of motivation for PA with PA level and 95%CI are calculated for each type of motivation as well. Intrinsic motivation ( $\hat{\beta }$ = 0.31; 95%CI 0.26–0.49), amotivation ( $\hat{\beta }$ = 0.16; 95%CI 0.10–0.34) and identified motivation ( $\hat{\beta }$ = 0.12; 95%CI 0.02–0.26) are those which, respectively, contribute the most to increase the PA level. If we look instead at the link between RAI continuum and PA level, we see that for an increase of one unit of RAI, PA level increases of 0.13 unit in average [0.01–0.04].

**Table 3:** Beta coefficient and 95% confidence interval of the association between motivation for physical activity and physical activity level.

Model	Variables	PA level*		
		$\hat{\beta }$	95% CI	
1	Amotivation	**0.16**	**0.10**	**0.34**
	External	0.05	−0.05	0.16
	Introjected	0.04	−0.03	0.10
	Identified	**0.12**	**0.02**	**0.26**
	Intrinsic	**0.31**	**0.26**	**0.49**
2	RAI	**0.13**	**0.01**	**0.04**

Estimate with confidence intervals excluding the null are bold. Model is adjusted for age, sex and mother’s education. *PAQ-C and PAQ-A questionnaire. Scores are interpreted as follows: 1 and 2 = Inactive; 3 and 4 = Active; 5 = Very active. RAI: Relative Autonomy Index.

**Figure 2:**
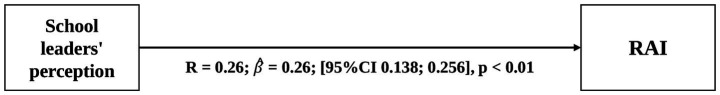
Associations between students’ perception of school leaders’ implication and relative autonomy index.

**Figure 3:**
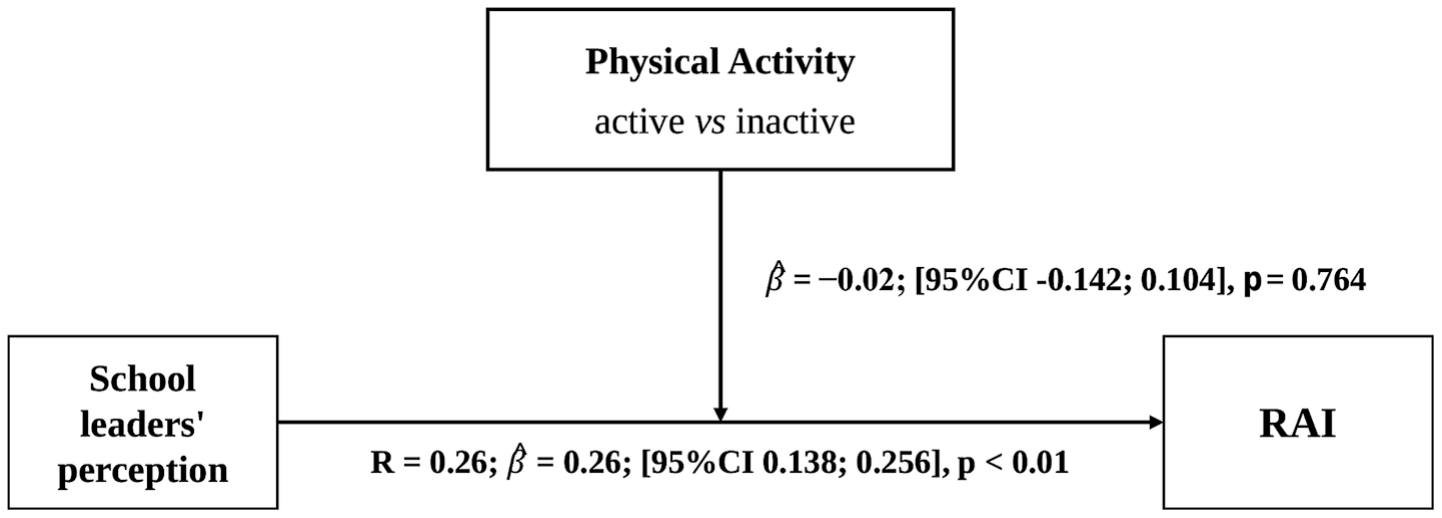
Moderation effect of physical activity level on the association between students’ perception of school leaders’ implication and relative autonomy index.

**Figure 4:**
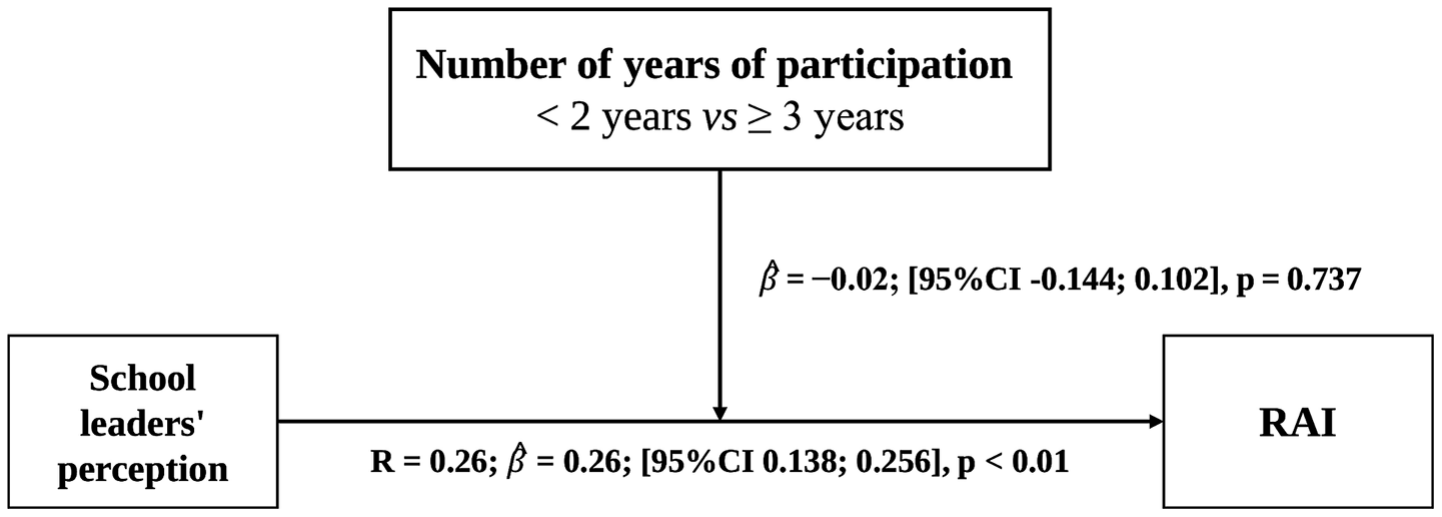
Moderation effect of the number of years of participation in GHP on the association between students’ perception of school leaders implication and relative autonomy index.

Direct association and moderation paths are shown in Figures 2–4. For the regression analysis, the predictor was “students’ perception of school leaders,” the outcome was “RAI.” The first hypothesized moderator was “Being active *vs* Inactive” and the second hypothesized moderator was “< 2 years of participation in the GHP *vs* ≥ 3 years.” Models were adjusted by covariables when applicable.

Model 1 shows a positive association between school leaders implication score and RAI [*R* = 0.26, 95% CI (0.138, 0.256), *p* < 0.01]. For a one score increase in the students’ perception of school leaders’ implication, the RAI is expected to increase by 0.26 unit, on average.

To infirm or confirm the hypothesis that PA level has an influence on the strength of the link between students’ perception of school leaders and RAI, a simple moderation analysis was performed. Specifically, we examined the moderating effect of the dichotomous variable PA (active = 0 *vs* inactive = 1) on the association between the students’ perception of school leaders score and RAI. By adding the information of the student’s PA level, simple moderation analysis was not statistically improving the model [Interaction term: $\hat{\beta }$ = − 0.02, (95%CI −0.142; 0.104)]. The association between students’ perception of school leaders score and RAI has not been enhanced by the addition of the moderator PA.

We further explore the moderation analysis to examine whether the number of years of participation in the GHP program (< 2 years = 0 *vs* ≥ 3 years = 1) moderated the relationship between school leaders’ implication and RAI. The interaction term between perception scores and years of GHP participation was not statistically significant [ $\hat{\beta }$ = − 0.02, (95%CI −0.144; 0.102)], suggesting that the effect of perception scores on RAI does not vary as a function of GHP participation duration. Although it was hypothesized that more years of GHP participation would strengthen the relationship between students’ perception of school leaders and RAI, this was not supported by the data. In other words, regardless of how long participants have been involved in the GHP program, their perception of school leaders’ implication did not have a significantly different association with RAI.

## Discussion

This study aimed to investigate the association between school leaders’ implication and motivation for PA in GHP participants, and to test whether this association is moderated by PA level (active *vs* inactive) or number of years of participation in the program (< 2 years *vs* ≥ 3 years); our findings partially supported these hypotheses, showing a significant positive association between school leaders’ implication and autonomous motivation (RAI), while neither PA level nor years of participation significantly moderated this relationship.

### Relationship between the type of motivation and PA

Results shown that three types of motivation for PA have contributed positively to PA level; intrinsic motivation, identified regulation and amotivation. However, intrinsic motivation shows the strongest association with PA level ( $\hat{\beta }$ = 0.31; 95%CI 0.26; 0.49). As mentioned in self-determination theory, intrinsic motivation, the most autonomous and effective form of motivation, drives an individual to perform an activity for the satisfaction it provides and not for the prospect of reward or punishment (Sarrazin et al., 2011; Alecu et al., 2025). It is important to encourage and to not interfere with intrinsic motivation with a reward, because studies show that perceiving a reward as controlling can lead to a decrease or even disappearance of intrinsic motivation (Sarrazin et al., 2011). Motivational climate also plays a major role in the perception of external influences. If school leaders are perceived as controlling rather than informative, they can hinder the need for autonomy and thus reduce intrinsic motivation. An important consideration in the results is the fact that almost all students in the program reported having autonomous motivation. It may be surprising, but it has happened in the past that students report high levels of motivation, even though teachers report dealing with demotivated students (Girard et al., 2023).

As shown in the Table 2, identified motivation (recognizing the personal importance of PA) has a weaker but significant association ( $\hat{\beta }$ = 0.12; 95%CI 0.02; 0.26) with PA level. Identified regulation is considered a self-determined form of extrinsic motivation that occurs when individuals recognizes the importance of the activity to achieve personal goals, so he/she participates voluntarily, even if it is not always pleasant (Ryan and Deci, 2000; Guay, 2022). At this moment, there is no pressure from the environment. It is the individuals that choose to commit for themselves to achieve meaningful personal goals. It can have a positive influence in the lasting hold of the behavior. Describing the benefits of PA on well-being and health during activities could therefore modestly contribute to the internalization of its practice. This can be easily implemented during activities, by example if leaders or participants talk about their own positive experiences related to PA. These experiences may help children and adolescents internalize the value of PA by linking it to personal goals. Students’ perception should be deepened and interventions aiming to boost PA should prioritize fostering self-determined forms, such as intrinsic motivation—for instance, by enhancing enjoyment, autonomy, and self-competence (Girard et al., 2023).

Table 2 brought also the fact that amotivation, typically negatively associated with PA (Faílde-Garrido et al., 2022; Surprenant et al., 2024), is surprisingly positively linked with increased PA level ( $\hat{\beta }$ = 0.16; 95%CI 0.10; 0.34). This finding may require a nuanced interpretation. A hypothesis could be made to explain that result. It is possible that students reporting amotivation are still being active due to external pressures or routine. In both cases, further qualitative research would help to clarify this paradox. As seen in Girard et al. (2023), focus groups and interviews could be more effective to identify and measure students’ motivation and perceptions.

In accordance with the previous results, students’ RAI has shown a statistically significant effect on PA level ( $\hat{\beta }$ = 0.13 per unit increase). However, the small magnitude suggests that composite indices like RAI may dilute the influence of specific motivational types (like intrinsic motivation). Indeed, each component is on the self-determination continuum but has its own particularities and adds information on the source of the motivation, the regulation and the level of autonomy satisfaction (Howard et al., 2017). Therefore, even if RAI is useful as a summary measure, it should not replace analyzes of individual motivational components in predicting behavior.

### Relationship between students’ perception of school leaders and RAI

After having tested the role of perceived school leadership implication in promoting autonomous motivation, the regression results suggests that students who perceive greater implication from school leaders tend to have more autonomous motivation toward PA [ $\hat{\beta }$ = 0.26; (95%CI 0.138; 0.256)], in review was consistent with previous study highlighting the positive influence of supportive school leadership on students’ motivation for PA (Vaquero-Solís et al., 2022). Indeed, a recent study found that elementary school students who perceived higher autonomy support from their physical education teachers exhibited greater autonomous motivation toward physical activity (Vaquero-Solís et al., 2022). This increased motivation was positively associated with their intention to be physically active and their actual physical activity levels. Our finding underscores the importance of the school environment—particularly leadership attitudes and behaviors—in shaping students’ motivational profiles. Supportive school motivational climate may reinforce basic psychological needs satisfaction among youth of autonomy, which are key drivers of RAI under Self-Determination Theory (Girard et al., 2023; Ryan and Deci, 2000). Taking this into account, school leaders’ training and institutional support could be leveraged to create environments that naturally foster autonomous motivation for PA among children and adolescents, as offered by the GHP.

### Moderating effects of PA level

Regarding the non-significant result for the moderation by PA level, the lack of a significant effect (*p* = 0.764) suggests that the relationship between students’ perception of school leaders and RAI is not contingent on students’ current PA levels. Indeed, being active or not does not influence the direct association between perception score and RAI. This could mean that perceptions of school support influence motivation regardless of whether students are currently active or inactive, making these perceptions a universally relevant factor. The direct pathway from perceived implication to motivation appears robust and should be a focus of school-based intervention efforts—regardless of students’ initial activity levels (Ourda et al., 2025). In this sense, the GHP can achieve PA motivation for all its participants, which is very encouraging for the continuation of the program.

Another important thought for this result is that if RAI is significantly associated with students’ perception of school leaders, and RAI is also associated with higher PA, then school climate might exert an indirect effect on PA behavior via its impact on motivational quality (Jung et al., 2021). However, given the relatively small RAI effect on PA ( $\hat{\beta }$ = 0.13), this pathway may be limited in explanatory power in the present study.

### Moderating effects of the duration of participation in GHP

The hypothesis that the duration of the participation in the GHP would modulate the effect of perception of school leaders’ implication on students’ RAI was not supported. Indeed, the effect of the direct association did not differ based on how long participants have been in the GHP program (< 2 years *vs* ≥ 3 years), as shown by a non-significant interaction (*p* > 0.737). This may suggest that the association between students’ perception of school leaders and RAI is relatively stable across different number of years of GHP involvement and that perceived school support influences motivation similarly across different levels of program exposure. It could reflect that even with more years of participation, the quality or consistency of support may not differ meaningfully between groups. This could also indicate that even early in the program, students develop strong perceptions of leadership support that remain stable over time. Otherwise, it could also reflect a similarity on how support is experienced across varying durations, pointing to a consistent quality of support provided to all students, regardless of how long they have been in the program. These results highlight the potentially immediate and sustained influence of perceived school leaders’ support on students’ motivation, irrespective of exposure time. School-based intervention programs need to focus not just on duration but also on the depth and quality of implementation (Ourda et al., 2025; Dowling and Barry, 2020). Future research should investigate whether there is an optimal window or threshold of exposure to such interventions rather than assuming “more is better.”

Taken together, the moderation analyses suggest that neither students’ current PA levels nor their length of participation in the GHP program significantly alter the impact of perceived school leadership implication on RAI. This consistency reinforces the idea that school leadership perceptions exert a stable influence on youth motivational quality, independent of PA status or program exposure length.

### Practical implications

Interventions should target motivation enhancement, particularly focusing on intrinsic factors such as enjoyment, autonomy, and perceived competence. School leaders should be actively involved in promoting a need-supportive motivation climate, as seen in the GHP model. For example, schools can implement student-centered PA sessions that allow for choice, variety, and progression based on individual skill levels, which has been shown to enhance autonomous motivation (Ryan and Deci, 2000). Leadership practices that include visible support for PA initiatives, regular communication about health goals, and shared decision-making with students can reinforce a supportive environment (Ntoumanis et al., 2021). Additionally, our baseline results suggest that tailoring PA programs based on students’ motivational profiles may enhance engagement and effectiveness. These strategies can be embedded in the regular curriculum to ensure sustainability and inclusivity.

### Strengths and limitations

The main strength of this study was the investigation based on a sample of participants in the same school-based intervention but in 48 different schools, which makes our results more representative. To the authors’ knowledge, this is the first study to directly examine the association between students’ perception of school leaders and motivation for PA in a school-based intervention setting. However, several limitations should be addressed. First, the cross-sectional design of the study limits the causal interpretation of the findings. Our future ongoing longitudinal study will confirm causal pathways. Second, our dataset only used online self-report questionnaires. Although the questionnaires were all validated with similar population, without a researcher present to clarify ambiguous questions, participants may misunderstand terminology or respond based on incorrect assumptions. Third, it is important to note that integrated motivation was not included in the questionnaire. This type of self-determined motivation falls just below intrinsic motivation on the Self-Determination Theory continuum. Including it might have helped explain why some participants were autonomously motivated to engage in PA—not necessarily for the enjoyment of the activity itself, but because it aligned with their personal values and identity (Sarrazin et al., 2011). The observed association between motivation for PA and actual activity levels suggests that encouraging individuals to integrate PA into their daily lives—and helping them understand its benefits—can effectively foster autonomous forms of motivation, which are the most strongly linked to higher PA levels. Fourth, while this study was grounded solely in Self-Determination Theory to focus on individual motivational processes, we acknowledge the relevance of broader socio-ecological frameworks. These perspectives could serve as valuable complementary approaches in future research aiming to explore contextual and environmental influences on youth motivation and physical activity engagement in greater depth. Finally, although we controlled for some confounding factors in our study, such as age, sex, mother’s education level and the number of years of participation in the GHP, several important potential confounding variables were still not measured. These include socioeconomic status (e.g., household income), parental PA levels, access to recreational facilities outside of school, and psychological factors such as self-efficacy. A few studies have suggested that these variables are significantly related to both the motivation for PA and participation in intervention programs among youth (Tandon et al., 2021; de Oliveira et al., 2024; Hosokawa et al., 2023; Cohen et al., 2025). For instance, youth from higher-income households or those with physically active parents are more likely to engage in PA (de Oliveira et al., 2024; Rhodes et al., 2020), while psychological factors may either facilitate or hinder sustained engagement in PA (Rhodes et al., 2020).

## Conclusion

The findings of this study highlight the important role of motivation in influencing PA levels among youth. Specifically, intrinsic motivation was the strongest predictor of PA levels. This underscores the importance of fostering internal enjoyment and personal relevance in promoting sustained PA among students. Furthermore, students’ current PA levels and their duration of participation in the GHP program did not significantly modify the relationship between perceived school leadership implication and RAI. The influence of school leadership perceptions on students’ motivational quality remains stable, regardless of their PA status or exposure length to intervention programs. These insights emphasize the value of cultivating supportive school environments and leadership practices that consistently promote autonomous motivation. Practically, this means school leaders should be actively involved in creating motivational climates—by endorsing student-centered activities, providing autonomy-supportive communication, and embedding PA opportunities into the school culture. Such efforts, exemplified by the GHP in Québec (Canada), can contribute to sustained youth engagement in PA. Future research should build on these findings using longitudinal designs to clarify causal relationships and refine practical strategies for broader implementation.

## Data Availability

The raw data supporting the conclusions of this article will be made available by the authors, without undue reservation.

## Footnotes

^1^ https://www.fondationsanteglobale.com/

^2^Note that this scale does not measure integrated motivation, which is the closest form of intrinsic motivation.

^3^These tools are widely used and are validated measures in the field of PA and motivation research, particularly among children and adolescents, which aligns well with our study population and objectives.
